# MicroRNAs in Prion Diseases—From Molecular Mechanisms to Insights in Translational Medicine

**DOI:** 10.3390/cells10071620

**Published:** 2021-06-29

**Authors:** Danyel Fernandes Contiliani, Yasmin de Araújo Ribeiro, Vitor Nolasco de Moraes, Tiago Campos Pereira

**Affiliations:** 1 Graduate Program of Genetics, Department of Genetics, Faculty of Medicine of Ribeirao Preto, University of Sao Paulo, Av. Bandeirantes, Ribeirao Preto 3900, Brazil; danyel.contiliani@usp.br (D.F.C.); yasminar@usp.br (Y.d.A.R.); vitor.moraes@usp.br (V.N.d.M.); 2 Department of Biology, Faculty of Philosophy, Sciences and Letters, University of Sao Paulo, Av. Bandeirantes, Ribeirao Preto 3900, Brazil

**Keywords:** microRNA, miRNA-based therapeutics, miRNA-based diagnostics, neurodegenerative, prion disease, RNAi, scrapie

## Abstract

MicroRNAs (miRNAs) are small non-coding RNA molecules able to post-transcriptionally regulate gene expression via base-pairing with partially complementary sequences of target transcripts. Prion diseases comprise a singular group of neurodegenerative conditions caused by endogenous, misfolded pathogenic (prion) proteins, associated with molecular aggregates. In humans, classical prion diseases include Creutzfeldt–Jakob disease, fatal familial insomnia, Gerstmann–Sträussler–Scheinker syndrome, and kuru. The aim of this review is to present the connections between miRNAs and prions, exploring how the interaction of both molecular actors may help understand the susceptibility, onset, progression, and pathological findings typical of such disorders, as well as the interface with some prion-like disorders, such as Alzheimer’s. Additionally, due to the inter-regulation of prions and miRNAs in health and disease, potential biomarkers for non-invasive miRNA-based diagnostics, as well as possible miRNA-based therapies to restore the levels of deregulated miRNAs on prion diseases, are also discussed. Since a cure or effective treatment for prion disorders still pose challenges, miRNA-based therapies emerge as an interesting alternative strategy to tackle such defying medical conditions.

## 1. Introduction

### 1.1. Prion: An Unconventional Infectious Agent

Prion (pronounced *pree-on*) is an atypical etiological agent composed solely of a misfolded protein—(proteinaceous infectious particle [1]), which affects mammals causing a group of slow, progressive, neurodegenerative, lethal, untreatable disorders known as transmissible spongiform encephalopathies (TSEs). Historical documentation of prion diseases dates back nearly three hundred years when a disorder referred to as *scrapie* was reported in sheep [2] and later in goats. Other TSEs include bovine spongiform encephalopathy (BSE, also known as mad cow disease) in cattle; chronic wasting disease (CWD) in deer, elk, and moose; exotic ungulate encephalopathy (EUE) in nyala, oryx, and greater kudu; and related encephalopathies in camel, mink, and cat. In humans, prionic disorders include Creutzfeldt–Jakob disease (CJD), Gerstmann–Sträussler–Scheinker syndrome (GSS), fatal familial insomnia (FFI) and kuru (reviewed in [2,3]).

**Figure 1:**
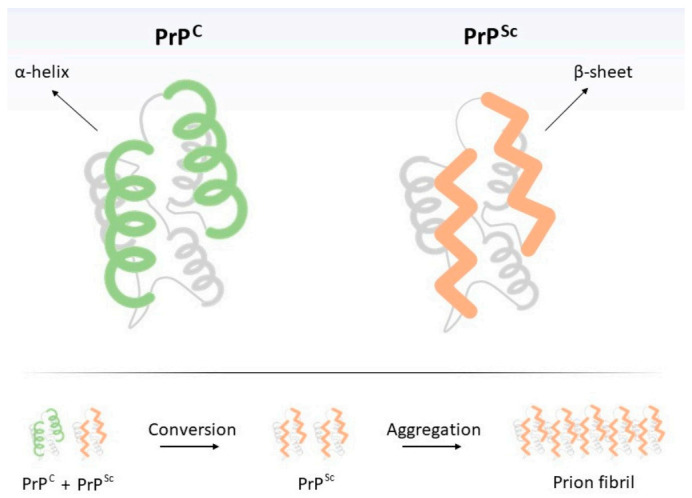
Structural differences between cellular (PrP^C^) and pathogenic (‘scrapie’, PrP^Sc^) prion proteins. Transition from PrP^C^ to PrP^Sc^ may occur spontaneously (sporadic form), due to the interaction of PrP^C^ with exogenous PrP^Sc^ (transmissible form) or it may result from a mutation in *PRNP* gene (inherited form). Below, PrP^Sc^ replication scheme. Intriguingly, since PrP^Sc^ is resistant to digestion by proteinases, ingestion of PrP^Sc^-contaminated food by humans (or animals), even if they do not possess mutations on *Prnp* gene, may result in the neurodegenerative disorder [8,9], thus justifying its transmissible nature. Finally, individuals may also develop transmissible spongiform encephalopathies (TSEs) in the absence of mutations or exposure to PrP^Sc^, resulting in the sporadic form of the disease, which is much more frequent than the hereditary form. Such situations seem to result from the PrP^C^-to-PrP^Sc^ spontaneous conformational change [10]. Briefly, prion diseases are unique by the fact that (i) their etiological agent is deprived of nucleic acid and (ii) they may be genetically inherited, acquired, or spontaneous.

It is perplexing to observe that, opposed to other conventional pathogens (viroids, viruses, bacteria, fungi, and parasites) which are extrinsic to the host, the misfolded prion protein has an endogenous origin. The natively folded version of the prion protein, named cellular prion (PrP^C^), is a cell-surface glycoprotein encoded by the endogenous mammalian gene *Prnp*, which is present in all vertebrates [4] and highly expressed in the brain, and at lower levels in most other tissues. Its precise organismal/cellular function is not clear, although many roles have been proposed, including stem cell renewal [5], memory formation, and myelin homeostasis [6]. It is soluble, protease-sensitive and its tertiary structure is rich in alpha-helices (Figure 1). However, missense mutations on the *Prnp* gene typically result in an insoluble, protease-resistant, and beta-sheet-rich folding version (the pathological, misfolded ‘scrapie’ version—PrP^Sc^) [7]. This, in turn, is non-functional and tends to form intra- and extracellular aggregates, which are possible causes of neuronal death. Therefore, TSE’s core element is, in fact, an incorrect version of a mammalian protein, and thus can be genetically inherited.

It is suggested that the physical interaction between PrP^C^ and PrP^Sc^ (as a monomer or a fibril) suffices for the conversion of the first into the second [10]. Thus, it is interesting to highlight that pathogenic prion (PrP^Sc^) amplification is not directly dependent on nucleic acids as all other known classes of pathogens. PrP^C^ may be interpreted as a proteinaceous substrate (rich in alpha-helices) on which the pathogenic misfolding (rich in ß-sheets) may spread. Therefore, pathogenic prion multiplication is an event of *structural information amplification*, rather than DNA or RNA replication. Nevertheless, since PrP^C^ synthesis is dependent on translation, transcription, and ultimately on a DNA sequence, PrP^Sc^ replication is indirectly dependent on DNA.

Notoriously, in the last decade, a series of discoveries have evidenced that the ‘prion principle’ may not be limited to the prion protein itself, but rather to many other mammalian (for example, a-synuclein and ataxin) and fungal (Sup35, Ure2; reviewed in [11]) polypeptides which are able to have a prion-like behavior (“prionoids”; [6]). These proteins present two structural conformations, the first acting as a template to the second, which is aggregation-prone, pathogenic and able to amplify its structural conformation. Many of these mammalian proteins are associated with neurodegenerative disorders, such as amyloid precursor protein in Alzheimer’s disease (AD), ataxin in amyotrophic lateral sclerosis (ALS), huntingtin in Huntington’s disease (HD) and a-synuclein in Parkinson’s disease (PD) (reviewed in [12]).

### 1.2. MicroRNAs: An Additional Layer of Gene Regulation in the Cell

**Figure 2:**
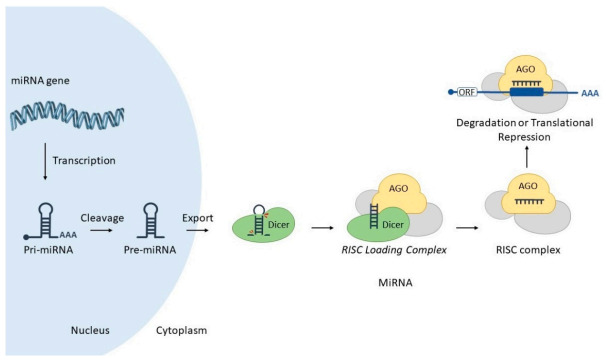
MicroRNA biogenesis and mode of action. A nuclear-encoded miR gene is transcribed into a pri-miRNA, which is cleaved by Drosha enzyme resulting in a pre-miRNA. In turn, this molecule is exported to the cytoplasm via Exportin-5. Dicer enzyme processes pre-miRNA into miRNA duplex, and associates into RISC loading Complex (RLC). This step allows the transfer of the miRNA from Dicer to Argonaute (AGO). Finally, miRISC complex interacts with target mRNA, promoting its degradation or translational blockade. Image includes elements from the Servier Medical Art (http://smart.servier.com/ accessed on 17 June 2021), licensed under a Creative Commons Attribution 3.0 Unported License (https://creativecommons.org/licenses/by/3.0/) (accessed on 23 April 2021). Detailed miRNA canonical biogenesis encompasses several steps. Briefly, a miR gene is transcribed by RNA polymerase II into a long, polyadenylated, 5′ capped molecule denominated primary miRNA (pri-miRNA), which is processed by enzyme Drosha into a precursor miRNA (pre-miRNA). In turn, this molecule is exported to the cytoplasm via Exportin-5/RanGTP complex, where it is further processed by enzyme Dicer into a miRNA duplex (guide and passenger strands). Dicer and miRNA duplex associates with other proteins to form the RISC loading complex (RLC), which allows the transfer of miRNA from Dicer to Argonaute protein (AGO), thus forming miRNA-induced silencing complex—miRISC (Figure 2) (reviewed in [15]).

MicroRNAs (miRNAs) are small (18–25 nucleotides) RNA molecules produced by the cell via the transcription of corresponding microRNA-encoding genes (miRs). MiRNAs are non-coding transcripts responsible for modulating the expression of other genes post-transcriptionally, via interaction with partially complementary sites at corresponding target RNAs. Such hybridization is mediated by a special ribonucleoprotein complex (miRISC), leading to targeted gene downregulation via RNA cleavage, deadenylation or translational inhibition (Figure 2) (reviewed in [13]). MiRNAs may also act through other mechanisms, such as transcriptional control (reviewed in [14]).

In general, miRs are intrinsically pleiotropic since one miRNA may interact with hundreds of different target RNAs, while one target RNA may also be regulated by different miRNAs. Virtually all cellular processes have been shown to be modulated by miRNAs, and since their discovery, they have been acknowledged as a new level of gene regulation, allowing the fine-tuning of genetic expression. 

In this review article, we will focus on the known cross-talk between classic prion diseases, associated with mammalian PRNP protein with miRNAs. Additionally, we will also outline the potential uses of these non-coding RNAs in unraveling key aspects of prion pathology, as well as the development of miRNA-based therapies. Although other disorders have recently been proposed to have prion-like etiological agents (AD, HD, PD, and ALS), they will not be the core of this review, since they involve several other genes and, consequently, hundreds of associated miRNAs.

## 2. Interplay between miRNAs and Cellular Prions in Health

Since both prion protein and microRNAs are encoded by the mammalian genome, both of them are subjected to many levels of expression control, as well as inter-regulation. Here we will briefly present some aspects of such molecular interactions between both actors.

### 2.1. miRNAs That Regulate Prion Expression

The identification of endogenous miRNAs able to downregulate prion protein has important implications for potential therapies, since decreasing PrP^C^ hinders PrP^Sc^ amplification. Accordingly, Pease and colleagues (2019) [16] decided to investigate, in vitro, which endogenous miRNAs are able to modulate prion protein. They constructed a sophisticated high-throughput arrayed screen, using a genome-wide miRNA library (a total of 2019 miRNA mimics), reverse transfection, and time-resolved fluorescence resonance energy transfer. They found 13 miRNAs able to directly regulate PRNP protein biosynthesis by binding to the 3′ untranslated region (3′UTR) of its mRNA, resulting in its degradation. The other four miRNAs (miR-124-3p, miR-192-3p, miR-299-5p, and miR-376b-3p) promoted changes in PRNP protein, however, probably by interacting with indirect regulators of PrP^C^.

Information on miRNA-mRNA interaction may also come from animal (in vivo) studies. For example, in order to understand the involvement of PrP^C^ accumulation in diabetic dementia-like pathology, Kalani et al. (2017) [17] assessed miRNAs expression via RT-qPCR array in brain cells of diabetes mellitus (db/db) mice (knockout for leptin receptor). Unlike the db/m control group, the analyses indicated underexpression of miRNA-146a (miR-146a) in db/db genotype. In addition, sequence analysis showed that miR-146a is able to bind to a conserved domain in the murine *Prnp* gene. Taken together, their data suggested that miR-146a modulates PRNP, and its downregulation in db/db diabetic mice explains PrP^C^ accumulation, previously known to be associated with the dementia-like phenotype.

In this context of miRNA-mediated post-transcriptional regulation, the identification of nucleotide variations within target sequences can provide indirect explanations for specific phenotypes. Given the strict requirement of high complementarity between a miRNA seed sequence and the 3′UTR portion of a target mRNA, nucleotide mutations within the seed sequence are typically detrimental to miRNA-mRNA interaction, hence directly affecting post-transcriptional gene regulation mechanisms. Interestingly, Zhao et al. (2016) [18] had reported that, compared to cattle, buffaloes have significantly lower PRNP protein abundance in the obex tissue, but significantly higher PRNP mRNA expression. To investigate whether this inconsistency was due to post-transcriptional regulation, Zhao and colleagues (2017) [19] decided to annotate the 3′UTR region of buffalo Prnp gene by 3′ rapid-amplification of cDNA ends, and sequencing. After comparing the differences in Prnp 3′UTR between cattle and buffalo, they found a total of 92 fixed differences, including four remarkable among them: two buffalo-specific insertion sites (a 28 base pairs insertion and an AG insertion in buffalo 3′UTR of PRNP, g.970-997 and g.1088-1089, respectively) and two substitutions (g.1007-1008 TG→CC) [19]. Additionally, the authors also performed luciferase reporter assays showing the direct interactions of five miRNAs (miR-125b-5p, miR-132-3p, miR-145-5p, miR-331-3p, and miR-338-3p) in the fixed differences sites in buffalo 3′UTR PRNP, and validated such downregulations by RT-qPCR.

### 2.2. Prion Protein Affecting miRNAs’ Expression

Some studies have revealed that a fraction of prion proteins remain in the cytoplasm, but their function in this cellular region was not clear. Although some researchers had found evidence that this cytoplasmic localized prion protein (cyPrP) is toxic to the cell, others had not. In order to shed light on this issue, Beaudoin and colleagues (2009) [20] created truncated versions of PrP, which tended to localize in the cytoplasm, forming a perinuclear ribonucleoprotein complex (RNP). They found that this complex was comprised of mRNAs, proteins (Dicer, Dcp1a, DDX6, SmB/B’/N), and non-coding RNAs (rRNA; U1 snRNA and microRNAs—miR-122a, miR-21, and let-7a). This structure resembled the ‘chromatoid body’, an RNA granule possibly involved in post-transcriptional gene regulation, typically found in germ cells and planarian stem cells and neurons. Their observations shed light on a possible role of PRNP in the cytoplasm, as well as suggested that potential interactions between prion protein and miRNA machinery in this perinuclear RNP may impact miRNA biogenesis.

Different studies have proposed diverse roles for prion protein, such as cell signaling, adhesion, and differentiation [21]. Based on evidence that PrP^C^ participates in neuronal differentiation [5,22,23], Shi and colleagues (2016) [24] decided to investigate whether prion protein could act as a modulator of this process in adipose-derived stem cells (ADSCs), an interesting source of stem cells. They submitted ADSCs to neuronal induction with IBMX (a competitive non-selective phosphodiesterase inhibitor), and through a series of experiments, the authors observed that PRNP protein contributes to the upregulation of miRNA-124 during this differentiation process in mouse ADSCs. They also report that this microRNA targets the 3′UTR of small C-terminal domain phosphatase 1 (SCP1) mRNA, an important anti-neural factor, thus prompting neuronal differentiation.

**Figure 3:**
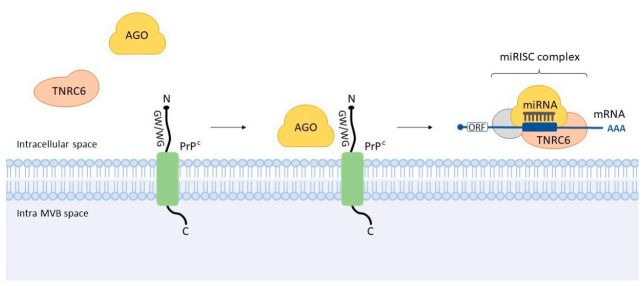
Interaction between cellular prion and Argonaute. It was observed that GW/WG motifs present in the N-terminal ‘octarepeat’ domain of PrP^C^ interact with the AGO, thus facilitating its interaction with TNRC6. This event seems to promote the assembly or stabilization of miRISC complex—an example of the interplay of prion protein and miRNA machinery. MVB: multivesicular body.

Interestingly, previous studies have evidenced putative links between prion protein and miRNA pathways. For example, via a protein-protein interaction screen, Satoh and colleagues (2009) [25] showed that recombinant PrP^C^ binds AGO1 (a key protein in the RNA interference and miRNA machineries). In turn, Spielhaupter et al. (2001) [26] revealed that PrP^C^ interacts with Eri3 (enhanced RNA interference-3; originally named “prion interactor 1”), which associates with Dicer and is directly related to RNA silencing in the model species *Caenorhabditis elegans*. Despite these findings, the molecular and cellular events concerning the transition of AGO from the ‘RISC-Loading Complex’ (RLC; containing Dicer) to miRISC remained unclear. Interestingly, although most prion protein is exposed to the extracellular milieu, one endogenous transmembrane PrP^C^ variant presents its N-terminal ‘octarepeat’ domain in the cytoplasm. Based on this fact and other evidence, Gibbings and colleagues (2012) [27] set out to interrogate this cellular process. They demonstrated that PrP^C^ interacts with AGO via GW/WG motifs present in the octarepeat domain and that it promotes the assembly or stabilization of miRISC complex (containing TNRC6 protein and targeted mRNA) (Figure 3). They also revealed that downregulation of diverse miRNAs targets necessitates PrP^C^.

## 3. Interplay between miRNAs and Prions in Disease

### 3.1. Dysregulated miRNAs in Prion Diseases

MiRNAs are crucial musicians in the molecular orchestra of the cell, and their expressions are dysregulated under stress conditions, as well as in several diseases [28]. Thus, many research groups have investigated miRNA expression profiles in patients diagnosed with prion diseases [29,30,31,32] as well as in animal models, such as (i) scrapie-infected murine models [33,34,35,36,37,38], (ii) SMB-S15 cells [36], (iii) scrapie-infected murine neuroblastoma cells [39], (iv) GSS-infected murine hypothalamic GT1-7 cells [40,41], (v) sporadic CJD (sCJD) mouse model [31], (vi) BSE-infected macaques [29], (vii) scrapie-infected sheep [42] and hamsters [37], and (viii) CWD-positive elks [37]. Moreover, temporal miRNA expression studies have highlighted the phase-specificity of miRNAs and their changing patterns throughout the progression of the disease [31,34,35,43].

Saba et al. (2008) [33] presented, for the first time, the global expression analysis of mature miRNAs in murine brains during prion disease. In their study, mice were intracerebrally inoculated with scrapie strain 22A, and miRNA expression was determined via microarray and RT-qPCR procedures. They observed 15 deregulated miRNAs, of which an important upregulation was seen on seven (let-7b, miR-128, miR-139-5p, miR-146a, miR-320, miR-328, and miR-342-3p) and a significant downregulation on two (miR-337-3p and miR-338-3p). Interestingly, the authors found several potential targets for these miRNAs through computational analyses, 119 of which had already shown to be dysregulated in murine prion disease models. They also conducted a computational-biochemical (*in silico* target prediction/dual-luciferase reporter assay) integrative approach to validate miRNA targets and found potential targets involved in signaling pathways related to cell death, synapse function, and neurogenesis [33]. This study showed an attractive approach for exploring miRNA expression profiles in prion-affected brains.

Nevertheless, the cellular and functional complexity of the brain is one of the major bottlenecks for an accurate interpretation of whole-brain gene expression patterns. The heterogeneous composition of the brain tissue, which includes different neuronal and supporting cell types (e.g., astrocytes, oligodendrocytes, and microglia), may mask temporal gene expression changes associated with prion-affected neurons. To address this challenge, Majer and collaborators (2012) [34] used laser capture microdissection technology to isolate neurons from the CA1 hippocampus region of scrapie-infected and control mice. Using microarray analysis, they were able to unveil a well-defined cluster of differentially expressed genes during the preclinical phase, including deregulated miRNAs miR-132-3p, miR-124a-3p, miR-16-5p, miR-26a-5p, miR-29a-3p, and miR-140-5p, which were validated by RT-qPCR. Finally, the authors suggested that the upregulation of genes (CAMK1, CAMK2D, RASGRF2, DOCK1) and miRNAs (miR-132-3p, miR-124a-3p, miR-29a-3p) might help neurons evoke a pro-survival response and stimulate dendrite remodeling mechanisms at the preclinical stage of prion disease [34].

Importantly, these miRNA alterations are shown to be dynamically related to the stage of prion disease [34]. In this regard, Toivonen and collaborators (2020) [38] decided to use *Prnp*-transgenic Tg501 mice to explore these temporal miRNA fluctuations in preclinical and clinical stages of mouse-adapted goat scrapie infection, comparing with age-matched, mock-inoculated controls. By carrying out small RNA sequencing of the cervical spinal cord, cerebellum, and plasma, they were able to identify significantly stage-specific deregulated miRNAs in preclinical- and clinical-stage animals (three and 23 miRNAs, respectively), which were predicted to be involved within known biological pathways, such as prion disease, extracellular matrix interactions, glutaminergic synapse, axon guidance, and transforming growth factor-beta signaling [38]. However, since the preclinical stage showed minimal changes, the authors suggested that most miRNA alterations are triggered by advanced prion-associated pathology, making it challenging to use them for clinical purposes, such as diagnostic biomarkers. 

Focusing on the advanced stage of scrapie disease, Gao et al. (2016) [36] carried out small RNA deep sequencing to unravel miRNA expression profiles in the brains of terminal-stage mice infected with scrapie agents 139A, ME7, and S15. As a result, a total of 57, 94, and 135 differentially expressed miRNAs were identified in 139A-, ME7- and S15-infected mice, respectively, whereas 36 miRNA alterations (up- and downregulations) were common to all infected groups—including two novel miRNAs, novel-miR-2 and novel-miR-20. Additionally, the Kyoto Encyclopedia of Genes and Genomes (KEGG) pathway analysis showed a total of 12 biological pathways shared among those three groups, including (i) olfactory transduction, (ii) metabolic pathways, and (iii) bacterial invasion of epithelial cells, which indicates a great similarity and coincidence of the potentially affected pathways in the terminal-stage brains infected with three different scrapie agents [36].

Whilst gene expression profiles were often explored in transmissible or genetically forms of prion pathologies, miRNA dysregulations associated with sporadic CJD (sCJD) remained poorly elucidated. In this regard, Llorens, Thüne and collaborators (2018) [31] carried out small RNA-Seq to investigate whether miRNA accumulation and its machinery (proteins Dicer, Drosha, microprocessor complex DGCR8, and Exportin-5) displayed differences in the two most affected brain regions (frontal cortex and cerebellum) within the two most prevalent sCJD subtypes (known as MM1 e VV2). Curiously, only 31% and 10% of the deregulated miRNAs were common to both subtypes in the frontal cortex and cerebellum, respectively, evidencing a considerable degree of divergence within sCJD. Alterations in miRNA machinery (except Exportin-5) were observed, however without regional/subtype-specificity, suggesting a complex interference. They also reported two shared dysregulated miRNAs (miRNA-877-5p and miRNA-323a-5p) in sCJD, AD, and dementia with Lewy bodies, evidencing a potential common underlying mechanism to these neurodegenerative disorders. Finally, they revealed that human brain and cerebrospinal fluid (CSF) miRNA profiles did not correlate, possibly due to the difference in the period of analyses—time of diagnosis for CSF versus post-mortem for brain tissue.

Interestingly, it was observed that miRNA-146a is upregulated in human brain cells infected with at least five distinct viral species, in diverse stress-induced human neuronal-glial primary cell co-cultures [44,45], as well as in a mouse model for scrapie [33] and in AD brain [46,47]. In order to investigate the profile of miRNA-146a in sCJD and GSS, Lukiw et al. (2011) [30] probed its expression via northern dot-blot and fluorescent miRNA array cluster analysis, and they observed its upregulation in both disorders. Thus, the role of this miRNA, which is abundantly expressed in murine and human brains, was expanded to other prion diseases, suggesting it is an integral part of the innate immune or inflammatory brain cell responses, with wide action. Moreover, this finding, along with previous studies, evidenced a shared underlying inflammatory response to neurological insults promoted by single-/double-stranded RNA/DNA viruses, AD, and prion diseases, when compared to healthy aged human controls.

### 3.2. Potential Contributions of miRNAs Dysregulation to Pathology

One important question in prionic disorders concerns the molecular/cellular changes that occur during the development of the disease, and how they are connected to the pathological findings. Evidence had shown an increase of miRNA-16 in hippocampal CA1 neurons during the presymptomatic phase of prion disease (in scrapie-infected mice), but how it might be involved in the pathology was not clear. Thus, Burak and colleagues (2018) [43] decided to conduct an experimental approach in which they overexpressed this miRNA using a lentivirus in mature hippocampal neurons of mice. Interestingly, they observed that neurons with higher levels of miR-16 displayed decreased neurite length and branching [43]. Moreover, via immunoprecipitation of Ago2-containing RISC complexes followed by microarray analysis, they identified some miRNA-16 targets including TrkB (NTRK2), MEK1 (MAP2K1), and c-Raf (RAF)—members of neurotrophin receptor-mediated MAPK/ERK pathway. Taken together, induction of miR-16 and the consequent downregulation of its targets might explain reduced neurite branching and length during the presymptomatic phase of prion disease.

As previously shown, miRNA-146a was reported as an inflammation mediator molecule in neurodegenerative diseases, including scrapie. Likewise, Saba and collaborators (2012) [48] evidenced through a series of different experimental approaches (microarray, RT-qPCR, gene overexpression/knock-down) that miR-146a is upregulated in the brain tissues of a murine model of prion disease, concomitantly with the onset of PrP^Sc^ deposits and activation of microglia. Authors suggested an additional function for miR-146a as an important regulator of microglial function by modulating the activation state during prion-induced neurodegeneration [48].

One of the first cytological manifestations in the neurodegenerative process concerns alterations in synaptic structural plasticity. For example, in prion disease, decreases in synapses and densities of the dendritic spine are observed in neurons of the cortex and hippocampus through the preclinical stage. The molecular processes underlying such events are still poorly understood. Interestingly, previous studies had reported miRNAs, pri-miRNAs, Drosha, and DGCR8 in neuronal dendrites and in synaptic fractions, where they are enriched [49]. Moreover, miRNA post-transcriptional regulation allows concurrent synaptic flexibility and stability, circumventing a delayed nuclear transcriptional gene modulation. Based on these key aspects, Boese and colleagues (2016) [35] hypothesized that miRNAs regulate synaptic protein synthesis in response to prion replication. They probed miRNA expression in synaptoneurosomes prepared from murine forebrain and hippocampus at two stages of prion disease. First, the preclinical period, when prion aggregates, gliosis, and dendrite loss are noticeable; and the clinical endpoint, when those first parameters are more prominent, accompanied by vacuolation [35]. They observed upregulation of miR-124a-3p, miR-136-5p, and miR-376a-3p during the preclinical phase. At the terminal stage, researchers found upregulation of several miRNAs (including let-7b, miR-142-3p, miR-143-3p, miR-145a-5p, miR-146a-5p, miR-150-5p, miR-320, miR-451a) previously reported to be dysregulated in brains of AD models as well as prion-infected mice. Also at the clinical phase, almost all members of the miR-200 family and miR-182 cluster decreased in abundance. Authors noted that several of those miRNAs target proteins associated with synaptic function and structural morphology, findings which may help understand prion pathology.

Interestingly, Jozef Nahalka (2019) [50] investigated an innovative, yet controversial concept of “protein-RNA recognition code” in the context of several neurodegenerative diseases (AD, HD, PD, ALS) and prion disorder. Essentially, the code is based on the idea of “reverse translating” an amino acid sequence or motif from a chosen protein, thus generating a nucleotide sequence that supposedly interacts with that polypeptide. According to the author, the code explains the interaction of (GGGGCC)n repeat in ALS with hnRNP H and SFPQ proteins, as well as between (CTG)n repeats in RNA, MNBL proteins, and RNA foci in myotonic dystrophy. More importantly, the code predicted that miR361 interacts with the spacer of the second and third α-helices of the prion protein, which seems to be important for prion conformational change, as well as with the ‘octapeptide repeat’ region. Accordingly, one study [35] in an animal model of prion disease reported deregulation of this microRNA, potentially evidencing a link between these two actors.

According to Montag and collaborators (2012) [39], disbalance in cholesterol homeostasis is related to the progression of neurodegenerative disorders, like prion diseases. Thus, in order to detect miRNAs involved in this process, they performed an ultra-deep RNA sequencing in scrapie-infected neuroblastoma N2a cells, including differential expression analysis against N2a-mock cells and subsequent validation by RT-qPCR. Interestingly, they found two downregulated miRNAs localized in a 5 Kb genomic cluster on the mouse X-chromosome—mmu-miR-351 and mmu-miR-542-5p-, that putatively bind to the 3′UTR of the cholesterogenic mouse genes involved in prion-induced dysregulation of cholesterol homeostasis Hgmcs11, Hgmcr, ldi1, CYP51, Ldlr, and Srebf2.

## 4. Potential miRNA-Based Diagnosis

**Figure 4:**
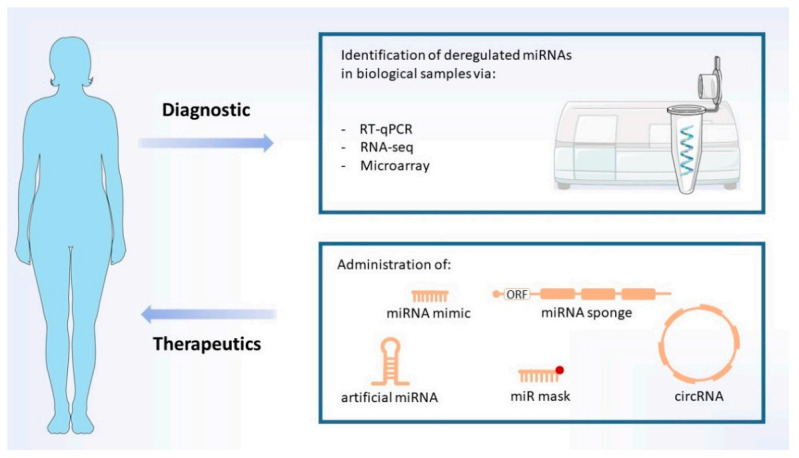
Diagnostic and therapeutics. Proposed diagnostic methods are based on analyses of prion disease-specific deregulated miRNAs (biomarkers). Potential therapies explore the use of several different miRNA-based strategies (indicated), which aim to restore the levels of down- and upregulated miRNAs. MiRNA mimics may be administered as single or double-stranded RNAs. Image includes elements from the Servier Medical Art (http://smart.servier.com/) (accessed on 17 June 2021), licensed under a Creative Commons Attribution 3.0 Unported License (https://creativecommons.org/licences/by/3.0/) (accessed on 23 April 2021).

Prion diseases may be diagnosed based on the presence of PrP^Sc^, for example, by the protein misfolding cyclic amplification (PMCA) [51], the amyloid seeding assay (ASA) [52], or the real-time quaking-induced conversion (RT-QuIC) [53]. However, those are highly specialized procedures, which are not available in every hospital or laboratory. On the other hand, diverse miRNAs are also altered during the course of prion disease, many of which might potentially be used for the development of alternative, simpler prion disease diagnostics. Such an approach is interesting since methods based on nucleic acid detection, sequencing, and/or amplification are very well established and might be rapidly adapted to any prion disease-specific set of miRNAs, in humans and animals (Figure 4). Following, some examples are reported. 

Due to this lack of a simple diagnostic test for detecting TSEs in animals, such as sheep and goats, Rubio and colleagues (2017) [42] analyzed a set of eight prion disease-associated miRNAs from the blood plasma of naturally scrapie-infected and healthy sheep, using RT-qPCR. They found two miRNAs, miR-342-3p and miR-21-5p, which were significantly upregulated only in classical scrapie sheep showing clear symptoms of the disease [42]. However, the authors cautioned that due to temporal changes of miRNA expression in TSEs, it is important to conduct similar experiments in preclinical sheep, thereby encouraging the development of alternative ways of diagnosis.

Using Illumina next-generation sequencing (RNA-Seq), Slota and colleagues (2019) [37] decided to perform a global analysis of the miRNA content of serum from 35 CWD-positive elks (at clinical and preclinical stages) and 35 CWD-negative elks, to determine differential miRNA expression profiles. Additionally, they also mapped conserved miRNAs from the sera of six experimental scrapie-infected, and six mock-infected hamsters, in order to compare miRNA expression profiles between species (elk and hamsters). For elk serum, a total of 21 miRNA biomarkers displayed altered expression in CWD-positive animals. On the other hand, a total of 35 differentially expressed serum miRNAs were found in scrapie-infected hamsters. Noteworthy, comparing elk and hamster altered miRNAs, they found 2 upregulated (miR-103a-3p and miR-107) and 4 downregulated (miR-99a-5p, miR-100-5p, miR-125a-5p, and miR-125b-5p) miRNAs that were shared between both infected species. Since most of these commonly altered miRNAs were among the best candidate CWD biomarkers, they may serve as future non-invasive diagnostic targets for cervid prion disease as well as in other species [37].

According to Norsworthy and colleagues (2020) [54], available blood tests are not able to discriminate sCJD from other neurodegenerative disorders, such as AD for example. Thus, they set out to investigate whether they could find a miRNA signature specific to sCJD in human blood samples. Using RNA sequencing, RT-qPCR, and two independent patient groups, they were able to validate the downregulation of three miRNAs (hsa-let-7i-5p, hsa-miR-16-5p, and hsa-miR-93-5p) and the upregulation of four of their targets (CCND3; CDKN1A; ZFP36; NAPL1L) [54]. They did not find any correlations of miRNA dysregulation with clinical parameters (age of onset, duration of disease, and MRC Scale score, for example) or disease progression. However, interestingly, this set of miRNAs was highly precise in discriminating sCJD from AD.

In order to confirm, in primates, the deregulation of miRNAs previously observed in murine prion disease models, Montag et al. (2009) [29] infected cynomolgus macaques (*Macaca fascicularis*) with brain homogenate from BSE-infected cattle and analyzed them. Animals were euthanized when three or more clinical signs were observed (myoclonus, loss of hand-eye coordination, apathy, and/or dehydration). Molecular confirmation of prion disease was performed via biochemical and immunohistochemical methods, which detected PrP^Sc^ aggregates in the brains of BSE-infected macaques. Assessment of miRNA differential expression (one healthy versus one diseased animal) was first conducted via microarray, on the *basis pontis* brain region. To validate their initial findings, the group performed RT-qPCR analyses on another group of animals (six BSE-infected and five non-infected macaques), which indicated upregulation of hsa-miR-342-3p and hsa-miR-494 in the brains of diseased macaques [29]. Finally, in a pilot study, they also confirmed hsa-miR-342-3p induction on two sCJD human patients, thus suggesting that this miRNA might be used as a biomarker for animal and human TSEs.

Polymorphisms in the locus ZBTB38-RASA2 have been associated with susceptibility to sCJD in the United Kingdom [55], while dysregulation of miR-146a has been reported in multiple sclerosis (MS), pro-inflammatory neurodegeneration and prion disease. Based on these findings, Gao et al., (2018) [56] investigated the correlation of two polymorphisms in ZBTB38-RASA2 and one in miR-146a with different aspects of sCJD or FFI in a Chinese population. Although they did not find any associations of the three polymorphisms investigated (ZBTB38-RASA2—rs295301; miR-146a—rs2910164 and rs57095329) with sCJD susceptibility; the single nucleotide polymorphism (SNP) rs295301 did correlate with the appearance of myoclonus, while rs57095329 with mutism and the positive of cerebrospinal fluid protein 14-3-3 in sCJD patients. Interestingly, rs57095329 was associated with FFI susceptibility. Such findings might be applied for specific diagnostic/prognostic tests.

Exosomes are small (~100 nm) extracellular vesicles of endocytic origin, enriched in cholesterol, phosphatidylserine, and specific proteins. They are cell-to-cell conveyors of miRNAs (mRNAs, proteins, and lipids) [57] from their originating cells through biological barriers. In order to investigate a potential link between exosomes and miRNAs, Bellingham and colleagues (2012) [40] assessed the RNA contents of such vesicles produced by neuronal cell cultures (mouse hypothalamic neuronal (GT1-7)) that simulates, the preclinical prion disease in vitro. Via small RNA deep sequencing, authors identified diverse transcripts including fragments of mRNAs and tRNAs, small nuclear RNAs, small nucleolar RNAs, small cytoplasmic RNAs, non-coding RNAs, silencing RNAs, retroviral RNA repeat regions, and miRNAs—known and novel ones. Importantly, they revealed that prion-infected neuronal cells released exosomes enriched with let-7b, let-7i, miR-21, miR-29b, miR-128a, miR-222, miR-342-3p, and miR-424, and depleted in miR-146 when compared with exosomes from non-infected cells. These data evidenced that circulating exosomes released during prion disease have a unique miRNA signature, with potential use in the development of diagnostic exams as well in the understanding of disease mechanisms.

Accordingly, other studies on exosome-packaged miRNAs also emphasized the feasibility of their use as non-invasive diagnostic biomarkers via blood sample analysis [58,59], since these brain-derived extracellular vesicles (EVs) may cross the blood-brain barrier (BBB) [60]. Thus, Cheng and collaborators (2021) [32] decided to investigate a set of differentially expressed EV miRNAs in sCJD patients, which had been previously reported to undergo dynamic expression changes in murine prion disease progression. Using RT-qPCR assay, they validated the expression of this set of serum EV miRNAs from patients with sCJD and controls. Since prion-associated EV miRNA biomarkers were highly sensitive (85%) and specific (66.7%) for sCJD prediction, these findings paved a way for an effective approach to detect prion-related diseases, including sCJD [32].

## 5. miRNA-Based Therapeutics

**Figure 5:**
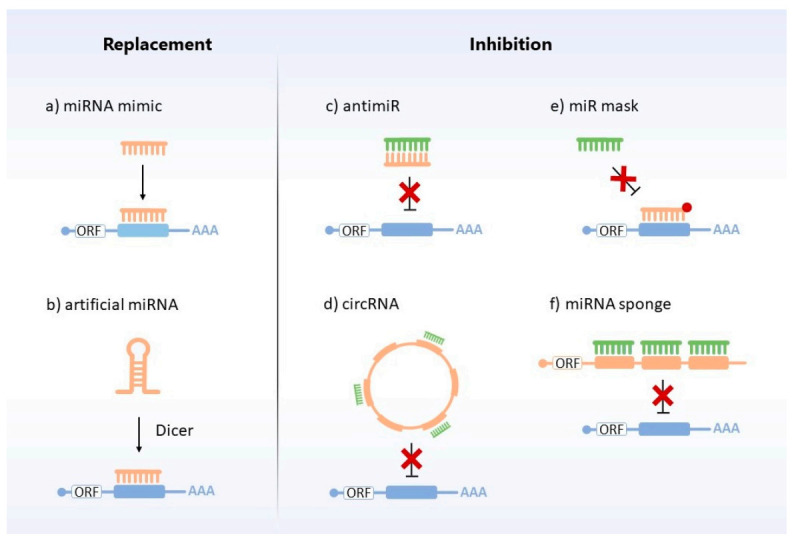
MiRNA replacement and inhibition therapies. Different strategies are used to restore miRNAs’ levels. Replacement approaches include: (**a**) chemically synthesized, mimic miRNA (orange), which targets the complementary mRNA (blue); (**b**) artificial miRNA (orange) which, after cellular processing, acts by binding to the target mRNA (blue) and degrading or repressing its translation. Inhibitors encompass: (**c**) antimiR (orange), which binds to the target miRNA (green) and makes it impossible to interact with the target mRNA (blue); (**d**) circRNA (orange), which sequesters the miRNA (green) from their target mRNAs (blue); (**e**) miR mask (orange), which blocks the action of the miRNA (green) by covering the binding site of its target mRNA (blue) without repressing it; (**f**) miRNA sponge (orange), which decreases the action of miRNAs (green) on their respective target mRNAs (blue), serving as a “trap” for miRNAs.

Normalization of the levels of miRNAs deregulated during prion diseases may be possible via different strategies. First, miRNA replacement approaches such as miRNA mimics and artificial miRNAs focus on increasing the intracellular/intra-organismal amounts of certain miRNAs which are downregulated throughout the course of the pathology. Conversely, miRNA inhibition strategies such as antimiRs, antagomiRs, and miRNA sponges aim at decreasing the levels of those miRNAs which are upregulated during the disease (Figure 5). Finally, a third group of concepts such as RNA interference, which resembles miRNA activity, may be used to suppress PrP^C^ mRNA, and thus hinder PrP^Sc^ amplification.

### 5.1. miRNA Replacement Approaches

A miRNA mimic is a synthetic single- or double-stranded RNA with perfect sequence identity to a natural miRNA. Although miRNA mimics have been used in vitro to validate putative prion mRNA-targeting miRNAs [16], to our knowledge, no replacement approaches have been tested in vitro or in vivo aiming to normalize deregulated miRNAs in (*PRNP*-linked) prion diseases to date. 

Nevertheless, proposals have been raised. For example, Goold and colleagues [61] centered their analysis on proteome stasis (or proteostasis), which is the balance between protein synthesis and degradation, needed to replace and remove denatured proteins, respectively. This aspect of cellular biology is affected in prion diseases, as an accumulation in PrP^Sc^ is observed. It has been suggested that pharmacological strategies that induce misfolded protein degradation mechanisms, such as the ubiquitin-proteasome system (UPS) and/or lysosomal proteolysis (including autophagic pathways) may help in the clearance of intracellular levels of PrP^Sc^. Such a concept is especially valuable in neurons due to their unique cytoarchitecture, extended longevity, and incapability to dilute toxic proteins via cell division [61]. In fact, several studies have demonstrated that induction of autophagy promoted PrP^Sc^ clearance and clinical benefits in animal models (reviewed in [62,63,64]). Thus, considering that miRNAs regulate a myriad of processes in cellular life, Shah and colleagues (2018) [65] proposed that therapies focused on the modulation of miRNAs involved in the control of autophagy processes might bring beneficial effects to neurodegenerative conditions caused by misfolded proteins. This approach might be based on the use of miRNA mimics that target negative regulators of autophagy, or anti-miRs that target miRNAs which themselves act as negative modulators of autophagic events. Although less tractable via conventional drugs, and a comparatively lower role on PrP^Sc^ clearance, UPS also poses as a potential system to be positively regulated by miRNA modulation. Such approaches remain to be tested.

Interestingly, miRNA replacement strategies have been investigated in other prionic diseases, as AD. In AD brains, several members of the miR-15/107 superfamily have already been reported to be misregulated, such as miR-16 [66,67], miR-15a/b [68,69,70,71,72], miR-195 [73,74], and miR-103/107 [75,76,77]. Given that some of these molecules associate with amyloid-beta (Aβ) production, Tau phosphorylation and regulation of BACE1 and amyloid precursor protein (APP) expression [69,74,78], such dysregulations may lead to the aggravation of AD pathology. In order to assess the feasibility of using miRNA mimics as a strategy to address this issue, Parsi and colleagues (2015) [79] decided to evaluate the effects of miR-16 mimics within wild-type (WT) mouse brains. For seven days, a dose of 50 µg/day of miR-16 mimic was administered by osmotic pumps in mice (treatment); whilst the control group received saline 0.9%. RT-qPCR analysis was carried out to evaluate the levels of miR-16 mimic in the brains of treated mice, thus showing significant increases in the hippocampus, cortex, striatum, and brainstem. Then, western blot analysis was performed for the quantification of APP, BACE1, Tau, and ERK1 in those four brain regions. Curiously, whereas region-dependent regulation of BACE1, APP, phosphorylated Tau, and ERK1 levels were observed, only APP and Tau showed significant reductions in mRNA expression (only in the cortex and brainstem, respectively) [79]. Another challenging point for the use of miRNA mimics as a therapeutic approach is the multiplex potential of a single miRNA to regulate several genes. In this regard, due to the well-described activity of miR-16 in inflammatory processes [80,81], Parsi and colleagues (2015) [79] screened for potential side effects related to brain inoculation of miR-16 mimic. Thus, RT-qPCR assays and proteomic analyses, followed by western blot validation, revealed a small number (~100) of downregulated genes, including inflammation markers (Gfap, for example) and other depleted proteins (α-Syn, Srrm2, GAPVD1, and TfR1) in mimic-treated mice [79]. Finally, these results suggest that miR-16 replacement therapy may be promising for AD and, perhaps, for other neurodegenerative diseases, such as PD [79] and classical prion diseases. However, this approach still needs to be supported by future experiments involving pharmacokinetic analyses of miRNA mimics in animal models of other neurodegenerative diseases.

### 5.2. miRNA Inhibitors and Others

Strategies to promote the loss-of-function of miRNAs have allowed scientists to experimentally validate several miRNA-mediated gene regulatory networks, including their key targets, and unravel their biological functions. One of such approaches uses morpholino oligonucleotides (MOs) [82]. When used for miRNA inhibition, these chemically modified molecules with optimized pharmacokinetic properties [83] are commonly referred to as antimiRs or antagomiRs, which are able to inhibit the function of specific endogenous miRNAs by binding to them via partial or total complementarity. In other words, when mature miRNAs are loaded on Argonaute proteins, antimiR molecules specifically pair to them [84], thus preventing miRNA-mediated mRNA regulation (competitive inhibition) [85]. Interestingly, MOs have already been explored as a therapeutic tool in some studies tackling human diseases [86,87,88,89], pointing to a novel form of treatment with miRNA-targeting drugs. 

Similar to the case of miRNA replacement therapies, no miRNA inhibition approach has been reported for classic prion diseases. Nevertheless, leveraging the plethora of biological data generated by high-throughput RNA sequencing techniques in recent years, MOs provide us with insights into the effects of spatiotemporal dysregulations in complex gene expression networks on other prion-like diseases. For example, RNA sequencing data of AD mice revealed that miR-331-3p and miR-9-5p are upregulated at the late stage of the disease, but not at the early one [90]. In this regard, this same research group decided to determine the effects of inhibiting these two miRNAs in AD mice. AntagomiR (treatment) and mock (control) groups were injected in the hippocampal region with the same volumes of 50 µM of antagomiR solution or PBS, respectively. After four weeks, memory and learning were evaluated by a range of behavioral analyses, including the Morris water maze and object location test. At the molecular level, western blot assays were carried out to evaluate Aβ accumulation, autophagy receptors (OPTN, SQSTM1, BECN1, and LC3B) in brain tissues of treated and control mice. Behavioral observations indicated that synergistic treatment of miR-331-3p and miR-9-5p antagomiRs might ameliorate memory loss and mobility/cognitive decline in AD mice. Moreover, compared with the control group, antagomiR-treated mice showed lower levels of Aβ along with higher levels of BECN1, OPTN, SQSTM1, and the ratio of LC3B-II:I, suggesting that miR-331-3p and miR-9-5p antagomiRs can reduce Aβ accumulation by enhancing autophagy activity [90]. Therefore, this study clearly shows how miRNA inhibition may be an upcoming strategy in clinical studies and applications in classic prion diseases in the future.

### 5.3. Targeting PrP mRNA

#### 5.3.1. Prnp-Targeting Artificial miRNA

Artificial miRNAs (amiRNAs) are synthetic genetic constructs that resemble a corresponding endogenous miRNA, however with some modifications in the region of the mature miRNA, that ensure complementarity to a specific mRNA target [91]. Considering the secondary structural similarity of amiRNAs with their endogenous precursor miRNAs, these molecules undergo natural cellular processing and, thus, act as post-transcriptional regulators [92]. 

For instance, Gallozzi et al. (2008) [93] designed a *Prnp*-targeting pre-miRNA expressing vector which was transfected into mouse-derived peripheral-neuroglial cells by the lipofectamine method. Protein extracts were analyzed by western blot 48 h after the transfection, revealing an efficient inhibition of murine PrP^C^ compared with control cells. Additionally, the authors also decided to create transgenic mice by micro-injecting the pre-miRNA construct into eggs, whose integration was confirmed via PCR. As a result, brain samples of three transgenic lines displayed (via western blot and RT-qPCR) a significant and homogeneous downregulation (as high as 80%) of murine *Prnp* gene, suggesting that amiRNA is an efficient mechanism to downregulate prion mRNA [93].

Based on the fact that prion diseases are fatal and untreatable, and that PrP^C^ is essential for the spreading of PrP^Sc^, Kang and collaborators (2018) [94] decided to investigate PrP^C^ suppression by an artificial dual miRNA (DmiR) and its effects in pathogenic prion replication in C2C12 myoblasts and primary mixed neuronal and glial cells culture (MNGC). Initially, they utilized a DmiR, which targets two regions (encompassing nucleotides 36-56 and 668-688) of murine PrP^C^ transcript, to investigate its efficiency in suppressing PrP^C^ expression. They observed, via western blot assay, that the DmiR strategy is effective in PrP^C^ suppression, and that it is more efficient than a single targeting strategy. Thereafter, they investigated whether PrP^C^ knockdown by DmiR resulted in a reduction of the pathogenic conformer accumulation. Via western blot, authors observed that the strategy reduced prion replication by 67.9% in C2C12 and by 61.5% in MNGC cells [94], validating DmiR as an interesting approach for prion diseases. 

#### 5.3.2. RNA Interference

RNA interference (RNAi) is a technology that promotes post-transcriptional gene silencing through the use of double-stranded RNA molecules [95]. Although RNAi itself is not a miRNA-based strategy stricto sensu, both RNAi and miRNA molecular pathways overlap considerably. RNAi is recognized as a suitable experimental technology for functional genomics and gene silencing-based therapeutic approaches [96], which may be an interesting method to modulate cellular or pathogenic prion protein.

In this concern, allele-specific RNA interference (ASP-RNAi) has been used as a therapeutic approach for the inhibition of a specific disease-associated allele. However, efficient discrimination between wild-type and mutant alleles is a central and intrinsic technical challenge to this strategy. Thus, Ohnishi and collaborators (2008) [97] proposed to incorporate a structural modification by replacing bases in specific regions of small interfering RNAs (siRNAs) and short hairpin RNAs (shRNAs) so that there would be the recognition of the mutant alleles, but not of wild-type one. For such, they selected three PRNP variants with amino acid substitutions (P102L, P105L, and D178N) associated with GSS and FFI. Then, they generated synthetic siRNAs with base substitutions against mutant alleles. Data suggested that the modifications resulted in better efficiency of ASP-RNAi, especially when performed in specific regions such as central position, seed region, and 3′ end of the sense-strand. The modifications of siRNAs at specific positions appear to improve the ASP-RNAi system due to the relationship of these regions with the cleavage and recognition of target RNA, and interaction with the RISC complexes.

### 5.4. General Concerns on miRNA Therapies

MiRNA replacement and inhibition have been assessed as therapeutic strategies to modulate microRNAs deregulated in diverse diseases. However, Ridolfi and Hanin Abdel-Haq (2017) [98] highlighted that off-target effects are one of the major concerns involving miRNA mimics and artificial miRNAs, it is, the fact that increasing the organismal concentration of a certain miRNA artificially may result in its interaction with secondary, unintended mRNA sequences [98]. Conceptually, the inverse problem may take place when using miRNA inhibitors. A second drawback is a possibility that high doses of synthetic miRNAs may overdrive (saturate) miRNA machinery, thus precluding the natural processing of endogenous miRNAs and, consequently, their normal biological functions. As an additional layer of complexity, both authors discuss the fact that some miRNAs display a fluctuating expression during prion disease progression, thus making it difficult to correctly fine-tune their expressions. 

Concerning the delivery vehicles of replacement/inhibition molecules, according to Ridolfi and Hanin Abdel-Haq (2017) [98], several systems are available—liposomes, polycationic polyethyleneimine-based nanoparticles, and viruses, however, they tend to be cleared by opsonins, complements, coagulation factors, endosomes, serum proteins and/or antibodies. Moreover, accidental proviral integration on the host genome’s proto-oncogenes may trigger tumor development. Finally, the BBB imposes a technical obstacle to prion disorders. Thus, an interesting strategy developed in recent years refers to exosomes, which have the advantage of possibly being better tolerated by the immune system, not phagocytized by the mononuclear phagocyte system, facilitated extravasation through vessel fenestrations and transit across the extracellular matrix. However, preserving the stability and effectiveness of miRNA/exosome during production, storage and application is still a challenge, especially in terms of cost to the patient. Other approaches involving transplantation of miRNA-overexpressing differentiated stem cells or the use of miRNA-binding proteins, like NPM1, to protect the RNA cargo are possibilities to be explored [98]. 

## 6. Circular RNA, Sponges, and Competing Endogenous RNAs

A circular RNA (circRNA) is a sequence of ribonucleic acid that forms a continuous loop, without 5′-3′ polarities or polyA tail. CircRNAs are resistant, stable, and widely-conserved structures that display specific spatiotemporal expression patterns. Although the biological roles of circRNAs are not fully understood, they may act as miRNA sponges, alternative splicing regulators, and parental gene expression modulators. The biogenesis of circRNAs is related to different mechanisms of splicing, involving the participation of RNA-binding proteins. CircRNAs tend to associate with miRNAs involved in the etiology of neurodegenerative diseases, including those caused by prions. For example, overexpression of the PrP^C^ results in the expression of circRNAs, such as ciRS-7/CDR1as, which are associated with miRNAs relevant to the neuropathological context [99]. Finally, due to the presence and abnormal expression of circRNAs throughout the development of prion disorder and other diseases, Qu and colleagues suggested the use of these molecules as diagnostic or predictive biomarkers [100].

An example of the role of a circRNA in a prion-like disorder—Alzheimer’s disease—was reported by Akhter (2018) [101]. To understand the molecular mechanisms involved in AD, he investigated the functions of a circRNA commonly described in brain tissue, ciRS-7/CDR1as. According to the author, evidence suggested that ciRS-7/CDR1as acts as a competing (or competitive) endogenous agent (ceRNA), binding to miRNA-7 without being degraded, and thus inhibiting the action of this miRNA [101]. The low level of CDS1as results in increased expression of miRNA-7, which negatively regulates UBE2A, an important protein for neurodegenerative disorders such as AD. UBE2A plays an important role in the context of eliminating toxic amyloid peptides, thus its downregulation is associated with AD. Due to their potential roles as important modulators of AD, circRNAs have been described as interesting markers for diagnosis, prognosis, and therapies.

## 7. MiRNAs in Other Prion-Like Diseases

Intriguingly, protein misfolding processes—exemplified by the prion protein—are also manifested in highly prevalent neurodegenerative diseases such as Alzheimer’s (AD) and Parkinson’s diseases (PD) [102], amongst 20 human diseases referred to as *protein misfolding disorders* (PMDs). In general, the misfolding and aggregation processes in PrP^C^-to-PrP^Sc^ conversion are shared in PMDs [103]—β sheet-rich oligomers forming amyloid-like aggregates by a seeding-nucleation mechanism [103,104,105]. For instance, AD is well-characterized by the misfolding and aggregation of amyloid-β (Aβ) and hyperphosphorylated tau proteins. Moreover, the pathological mechanisms of prion disease transmission are also likely to be mirrored in AD, since experiments with transgenic mice expressing human amyloid protein have shown to develop the specific abnormalities of an AD profile—accelerated Aβ-deposition in brain and proteolysis-resistant seeding-competent Aβ aggregates—when they are intraperitoneal- and/or intracranially inoculated with Alzheimer’s brain extracts [106,107,108,109,110]. The transmission of protein misfolding is also observed in Tau proteins (AD) [111], Lewy bodies (α-synuclein aggregates) in PD [112,113,114], which suggests the prion-like transmission behavior in PMDs (reviewed in [12]).

Under normal conditions, the central nervous system (CNS) is protected by the BBB and displays well-defined immune responses against threats—neuroinflammation. On the other hand, under pathological conditions these inflammatory processes become dysregulated—uncontrolled neuroinflammation—and the CNS undergoes elevated glial cell activation, BBB permeability, and peripheral immune cell infiltration. Ultimately, such dramatic changes lead to neurotoxicity and even neurodegeneration [115]. Interestingly, these inflammatory processes may be either promoted or suppressed by the activity of miRNAs, such as miR-155, miR-146a, miR-124, miR-21, and let-7, which can be either synergistic or antagonistic. Beyond the involvement of these miRNAs in conventional prion diseases (e.g., sCJD and GSS), they are also involved in the following neurodegenerative disorders: Multiple Sclerosis (MS), AD, and PD. Slota and Booth (2019) [116] depict how the aforementioned miRNAs play similar overlapping roles within multiple pathologies. For instance, miR-155 acts as a pro-inflammatory factor in a similar way in MS, AD, and PD [117,118,119,120,121,122,123,124,125]; likewise, miR-146a operates as an anti-inflammatory agent in MS, AD and conventional prion diseases [48,117,123,125,126]. Conversely, other miRNAs’ roles may be disease-specific, such as let-7 which has been identified as upregulated in AD, acting as a danger-associated molecular pattern (DAMP) for toll-like receptor 7 (TLR-7) [127]. Therapeutic strategies for suppressing pro-inflammatory miRNAs (via antagomiRs, antimiRs, and miRNA sponges) or inducing anti-inflammatory miRNAs (via mimics)—allied to optimal molecular delivery systems-, can be encouraged from these findings. Additionally, Slota and Booth (2019) also highlighted how the discovery of disease-specific miRNA signatures in circulating fluids (e.g., serum, blood, and plasma) might paves a way for the use as non-invasive biomarkers to circumvent the complicated diagnosis of symptom-overlapping neurological disorders (reviewed in [116]).

Multiple neurodegenerative diseases, such as AD, ALS, HD, PD, and conventional prion diseases share pathophysiological processes that enable us to pinpoint common molecular mechanisms across them, thereby favoring the development of novel therapeutic approaches. In the search of miRNA dysregulation patterns across neurodegenerative diseases, Juźwik and colleagues (2019) [128] decided to perform a non-biased systematic review on this topic (miRNA and neurodegenerative diseases). A total of 2318 significantly dysregulated miRNAs (validated by qPCR only) were retrieved from each of the 641 accepted manuscripts, but only seven individual miRNAs (downregulated—miR-9-5p, miR-21-5-p, miR-124-3p, miR-132-3p; upregulated—miR-146a-5p, miR-155-5p, miR-223-3p) and 1 miRNA family (miR-29 family) were considered as prevalent across neurodegenerative diseases [128]. Interestingly, the authors summarized the functional overlaps between these miRNAs within cellular pathways targeted by multiple miRNAs. For instance, miR-146a-5p, the miR-29 family, miR-124-3p, and miR-9-5p work synergistically to limit Aβ genesis. Likewise, NF-κB signaling, BBB maintenance, neurogenesis, autophagy homeostasis, and microglial activation pathways involve the functioning of multiple miRNAs. Additionally, the gatekeepers of nervous and immune systems, known as NeurimmiRs, comprise miR-9-5p, miR-124-3p, miR-132-3p, miR-146a-5p, and miR-155-5p [129]. Considering that miR-21-5p, the miR-29 family, and miR-223-3p are responsible for T-cell differentiation and activation, and neuronal function modulation, the authors also suggested including these miRNAs as novel NeurimmiRs, which also seem to be involved in robust immune responses in neurological disorders [128]. 

Finally, Brennan and others (2019) [130] performed a wide literature search on PUBMED and Google Scholar platforms in order to identify human body fluid miRNA biomarkers for AD, PD, MS, and familial and sporadic forms of ALS reported in articles published from 2011 to 2018. The result was a ‘knowledge base’ encompassing 72 different studies and 347 unique miRNAs considered statistically significant. Interestingly, the authors noted that the prion disease pathway is one of the most constantly pictured pathways in their study, with high statistical significance [130]. They also observed that most of these miRNAs were upregulated, evidencing that this pathway is probably repressed on those neurodegenerative diseases. Finally, the authors highlight that fifty-seven percent of all miRNAs reported in other articles as dysregulated in prion disease were also identified in their human body fluid-focused survey, for example, hsa-miR-146a, miR-26a, hsa-let-7i, hsa-miR-424, and hsa-miR-128 [130].

## 8. Perspectives

Prions stand as the singular class of pathogens deprived of nucleic acids, able to promote biological amplification of structural information, and which diseases may manifest in three totally distinct forms (inherited, sporadic or acquired). Two Nobel prizes have been awarded to researchers dedicated to prions—Daniel Carleton Gajdusek (1976) and Stanley Benjamin Prusiner (1997). On the other hand, although microRNAs had been largely ignored by molecular biologists for decades due to their extremely small sizes, they have risen as important elements of the genetic regulatory circuitry from the early 2000 s, when their widespread occurrence and roles were acknowledged. Similarly, a Nobel prize was awarded to the discoverers of RNA interference, which is closely related to the microRNA pathway—Andrew Zachary Fire and Craig Cameron Mello (2006); although a prize specifically dedicated to miRNAs is also possible in the future. Therefore, it is extremely likely that the intersection of both research fields will be largely prolific and synergistic in the upcoming years. Specifically, the putative role(s) of (mi)RNAs in the process of prion conversion [131] remains an interesting avenue of investigation.

Notoriously, both miRNAs and prions have been described as mediators of epigenetic inheritance—the heritability of phenotypes in the absence of changes in the nucleotide sequence of the corresponding gene (reviewed in [132]). In fungi, for example, non-disease causing prion-like elements have been reported to modulate cellular traits, thus posing as important players of fungal biology [133]. Thus, it is possible that prion-like “amplification of structural information” without protein aggregation may be a common and normal cellular mechanism of phenotypic plasticity in humans and (all) other species. Such a hypothesis, if confirmed, could represent an important change in our understanding of the flow of biological information within the organisms. Likewise, the frontier of the universe of small RNAs is constantly expanding [134], with new microRNAs species being identified uninterruptedly, as well as their molecular idiosyncrasies [135,136,137,138]. Thus, diverse still hidden aspects of miRNA-prion interplay and cross-regulation may come to light in the upcoming years. 

It is also interesting to highlight that several neurodegenerative diseases have been proposed, and shown to some extent, to have a prion-like mechanism—AD, HD, PD, ALS among others. These findings call the attention of the scientific community to investigate such conditions from a new and different perspective. Understanding them through the prion prism may help to shed light on the exquisite phenomenon of structural amplification and aggregation. Moreover, some studies on cancer have also identified deregulated miRNAs that are associated with prion pathways [139,140,141,142]. Likewise, those results not only unveil cryptic connections between prion and oncogenesis but might also help in the development of alternative treatment approaches. Finally, other related entities such as (i) the mnemon—“a protein that super-assembles in a controlled manner to encode a memory of a past event and segregates discreetly, being inherited asymmetrically at mitosis or kept in a specific subcellular compartment in nondividing cells” [143] and (ii) intrinsically disordered prion-like proteins [144] may be investigated in neurodegenerative conditions.

Due to the unique nature of its etiological agent, prion diseases remain a medical challenge without a cure. Strategies to control prion-like disorders may focus on (i) avoiding misfolding of cellular protein, (ii) targeted degradation of misfolded conformers, (iii) inhibition of aggregation and/or plaque formation, among others. However, according to Ridolfi and Abdel-Haq (2017) [98], drugs designed to decrease the amounts of misfolded polypeptides in neurodegenerative disorders have had limited success in clinical trials. Available treatments are restricted to a group of compounds that modulate some of the initial symptoms but are not able to hinder neurodegeneration. Nevertheless, since the pathogen has its roots in the host cell genome, endogenous mechanisms of gene regulation (like microRNAs) possibly have important impacts on disease susceptibility, onset, development, and severity. Therefore, even if researchers come to the conclusion that it is impossible to hinder PrP^C^-to-PrP^Sc^ conversion and/or aggregation through conventional drugs, miRNA-based therapies might still be effective in depleting cellular levels of PrP^C^, thus hindering some aspects of the disease. Speculatively, gene editing via CRISPR [145] might also be a strategy in the distant future, to replace wild-type, conversion-prone (susceptible) allele(s) with a conversion-resistant allele(s) [146]; considering all ethical issues are observed and followed. In this context, prion’s cellular role would not be compromised, yet disease should not be able to manifest, even in the advent of exposure to a contaminated source.
